# Supplementary material for: Epicardial Adipose Tissue Thickness Is Related to Plaque Composition in Coronary Artery Disease
Source: Diagnostics (Basel). 2022 Nov 17;12(11):2836. doi: 10.3390/diagnostics12112836 (PMC9689801; doi:10.3390/diagnostics12112836)

Supplemental Data

Supplemental Figure 1. The $maxLCBI_{4mm}$ value according to the range of EAT thickness

A group '1' represents for patients in the range of 0-25th percentile of EAT thickness; '2' for 25-50th percentile, '3' for 50-75th percentile, and '4' for 75-100th percentile. The average $\mathit{max}LCBI_{4mm}$ value increased as EAT thickness increased. *p*-value was obtained from ANOVA test. EAT, epicardial adipose tissue; $maxLCBI_{4mm}$, maximum lipid core burden index in any 4mm segment.

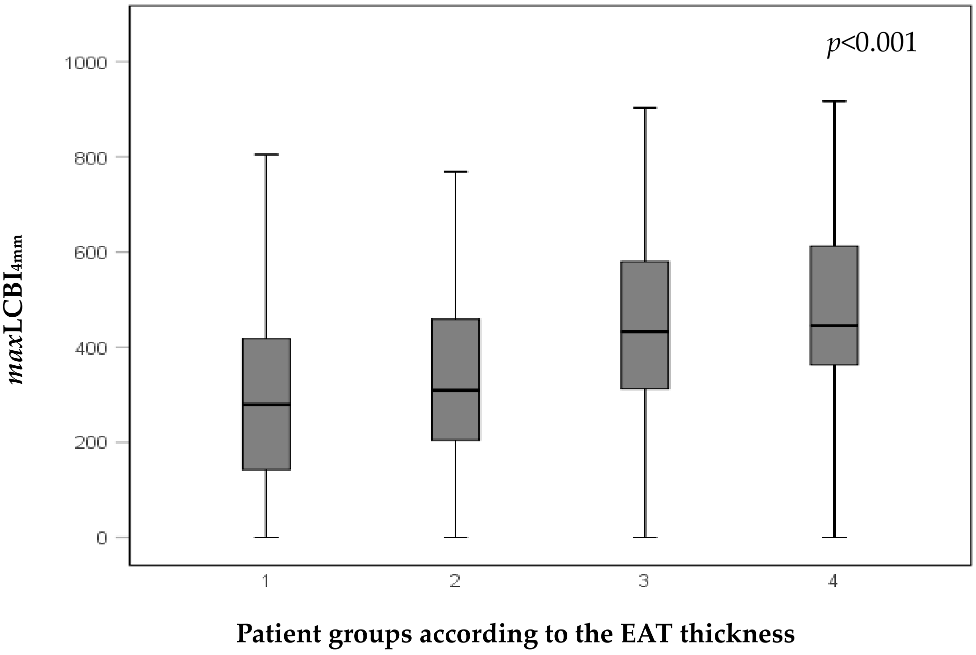

Supplement: Supplementary file 1 [file diagnostics-12-02836-s001.zip › diagnostics-2011787-supplementary.pdf]
